# Novel Mutations Identified in the Chinese Han Population with Keratoconus by Next-Generation Sequencing

**DOI:** 10.1155/2022/9991910

**Published:** 2022-02-10

**Authors:** Binbin Chen, Xiaoning Yu, Xin Zhang, Hao Yang, Yilei Cui, Xingchao Shentu

**Affiliations:** Eye Center of the Second Affiliated Hospital, School of Medicine, Zhejiang University, Zhejiang Provincial Key Lab of Ophthalmology, Hangzhou, Zhejiang Province, China

## Abstract

**Aim:**

To identify novel mutations in keratoconus (KC) susceptibility genes in the Chinese Han population.

**Methods:**

A total of fifty-two patients with primary KC were recruited. Blood samples were collected, and genomic DNA was isolated from peripheral blood leukocytes. The entire coding region, intron-exon junctions, and promoter regions of sixteen known KC susceptibility genes were screened with next-generation sequencing technology. All identified variants were further confirmed using the Sanger sequencing technology. The Sorting Intolerant from Tolerant (SIFT), MutationTaster, and PolyPhen 2 programs were used to predict the effect of amino acid substitution on protein.

**Results:**

After removing twelve known SNPs (single nucleotide polymorphisms) and three variants predicted to be harmless, nine novel mutations were identified in eight of the fifty-two patients, including c.455C > T:p.P152L in FNDC3B; c.3636_3637del:p.R1212fs in COL4A4; c.5015G > T:p.R1672L, c.3798dupA:p.P1267fs, and c.28G > A:p.A10T in MPDZ; c.1940C > T:p.P647L in DOCK9; c.127_128insGGC:p.Q43delinsRQ in POLG; c.3019G > A:p.V1007I in IPO5; and c.624 + 7− > A in TGFBI. All nine mutations in the patients with KC were heterozygote.

**Conclusion:**

This study enlarged the gene profile of KC and should be further confirmed by well-powered, genome-wide association studies (GWAS) of Han Chinese patients.

## 1. Introduction

Keratoconus (KC) is characterized by bilateral progressive corneal thinning and ectasia [1]. It typically begins in adolescence and progresses until the fourth decade of life [2]. KC is one of the most common causes of keratoplasty in the developed world [3]. According to epidemiologic studies, the prevalence of KC is approximately 8.8 to 54.4 per 100,000 individuals across the globe, depending on the diagnostic criteria of KC and population characteristics [3].

Despite the high prevalence of KC, the etiology of the disease is not well understood. Given the fact that KC is an irreversible disease, it is vital to identify patients with risk factors as soon as possible. Many studies have been performed to identify risk factors related to KC [4–7].

Genetic factors have been shown to contribute to the pathogenesis of KC, although the pathogenesis of many risk genes has yet to be determined [4]. Genome-wide association studies (GWAS) and candidate gene association studies have identified over 150 polymorphisms in more than 60 genes related to increased susceptibility to KC [8]. However, few studies have been conducted in Han Chinese patients with KC. To enlarge the genetic profile of KC in the Han population, this study was designed to screen the coding regions of NFIB (nuclear factor I B), LIG3 (DNA ligase 3), XRCC1 (X-ray repair cross-complementing 1), TF (transferrin), ZEB1 (zinc finger E-box-binding homeobox 1), NEIL1 (Nei like DNA glycosylase 1), FNDC3B (fibronectin type III domain-containing 3B), COL4A4 (collagen type IV alpha 4 chain), MPDZ (multiple PDZ domain crumbs cell polarity complex component), DOCK9 (dedicator of cytokinesis 9), TGFBI (transforming growth factor-beta induced), PARP1(poly(ADP-ribose) polymerase 1), POLG (DNA polymerase gamma), IPO5 (importin 5), FASLG (Fas ligand), and IL1B (interleukin 1 beta) genes in the Chinese population.

## 2. Methods

This study adhered to the tenets of the Declaration of Helsinki and the statement on human subjects by the Association for Research in Vision and Ophthalmology. It was approved by the ethics committee of the Second Affiliated Hospital, Medical College of Zhejiang University, Hangzhou, China. Informed consent was obtained from every participant.

Thirty male and twenty-two female KC patients, ranging from eleven to forty-three years of age, were willing to enroll in this study. All KC patients included in the study had negative family histories for KC. The diagnosis of KC was made based on clinical manifestations such as corneal stromal thinning, Vogt's striae, Fleischer ring, Munson's sign, conical protrusion of the cornea at the apex, and anterior corneal stromal scar using a slit-lamp microscope, and signs of videokeratography (localized, increased surface power, and/or inferior-superior dioptric asymmetry (Bausch & Lomb Surgical, Orbtek Inc., Salt Lake City, UT)). Any patient with KC with a coexisting allergy; atopy KC secondary to such causes as trauma, LASIK, or other refractive surgeries; or with Ehlers–Danlos syndrome, Down syndrome, osteogenesis imperfecta, or pellucid marginal degeneration were excluded from this research.

Blood samples (five ml) of the participants were collected in Vacutainer tubes (Becton Dickinson, Franklin Lakes, NJ) containing ethylene diamine tetraacetic acid. These tubes were preserved at −80°C before genomic DNA was extracted with a Simgen DNA Blood Mini Kit (Simgen, Hangzhou, China) according to the manufacturer's instructions. All patients with KC underwent mutation screening. For KC patients, all KC related genes, which were reported in the PubMed database before December 31, 2014, were screened with next-generation sequencing technology (Supplementary Material (available here)), which was based on targeted sequence capturing technology with the SureSelect Target Enrichment Kit (Agilent Technologies, Santa Clara, CA) and Illumina sequencing technology with the HiSeq sequencer (Illumina, San Diego, CA). All the screened variants were compared with the KC-related variants reported in the PubMed database before May 31, 2021.

To avoid false-positive results and to ascertain the significance of the mutations, the following mutations were subsequently confirmed using Sanger sequencing technology: (i) mutations with a minor allele frequency GWAS <0.1% (according to data from the May 2012 release of the 1000 Genomes Project and the Single Nucleotide Polymorphism Database); (ii) mutations that were absent from the results of the WES data acquired from 220 Han Chinese individuals without ocular abnormalities (from a commercial database provided by the Genesky Bio-Tech company); (iii) mutations that were never before reported in the PubMed database; and (iv) mutations that were implied as damaged to protein function according to bioinformatics software (as detailed further in this study).

All the coding regions (exons, intron-exon junctions, and promoter regions) of the sixteen genes were amplified with PCR using specific primer sequences. Three different PCR conditions were involved in this study:

1. Reaction conditions for fragments 1 and 6: the cycling program was 95°C for 2 min; 35 cycles × (96°C for 10 s and 68°C for 1 min); and 4°C forever
2. Reaction conditions for fragments 3 to 5: the cycling program was 95°C for 2 min; 11 cycles × (94°C for 20 s, 66°C minus 0.5°C/cycle for 40s, and 72°C for 1 min); 24 cycles × (94°C for 20 s, 60°C for 30 s, and 72°C for 1 min); 72°C for 2 min; and 4°C forever
3. Reaction conditions for fragments 2 and 7: the cycling program was 95°C for 2 min; 11 cycles × (94°C for 20 s, 62°C minus 0.5°C/cycle for 40 s, and 72°C for 1 min); 24 cycles × (94°C for 20 s, 56°C for 30 s, and 72°C for 1 min); 72°C for 2 min; and 4°C forever

The PCR products were isolated with electrophoresis and sequenced using the BigDye Terminator v3.1 Cycle Sequencing Kit (Applied Biosystems, Foster City, CA) on an Applied Biosystems ABI 3730 Sequencer Analyzer. Finally, the sequencing results were analyzed using the PolyPhred version and compared with the sequences in the NCBI (the National Center for Biotechnology Information) GenBank database.

The SIFT programs were used to predict the effects of mutations on proteins. Based on the theory of evolutionary conservation, amino acid substitution is considered damaging if the SIFT score is ≤0.05; otherwise, the substitution is tolerated. The other two kinds of bioinformatics software—MutationTaster and PolyPhen 2—were used to improve the accuracy of predictability. Both of the predicted scores were positively correlated with pathogenicity, and the significance level was set at 0.85. Only those that met the following criteria were recognized as harmful to the function of the protein: (i) SIFT score ≤0.05 and (ii) MutationTaster or PolyPhen 2 score ≥0.85.

## 3. Results

The entire coding region, intron-exon junctions, and promoter regions of all involved genes were screened. Twenty-four variants in NFIB, LIG3, XRCC1, TF, ZEB1, NEIL1, FNDC3B, DOCK9, COL4A4, POLG, MPDZ, IPO5, TGFBI, PARP1, FASLG, and IL1B genes were identified in fifty-two sporadic KC patients with next-generation sequencing technology. A total of twenty sequence variants were nonsynonymous single-nucleotide variants; two variants caused frameshift insertion; and two were located in the region of 2 bp around the splicing junction. These results are summarized in Table 1.

The following were excluded from the study: twelve known SNPs (single nucleotide polymorphism) (rs140030018, rs2271980, rs150679929, rs142213781, rs201974179, rs201101621, rs200630156, rs201256399, rs181860632, rs201477273, rs5602117, and rs200401035) and three single nucleotide variants (LIG3, c.1626G > T; ZEB1, c.2998G > C; and PARP1, c.114G > A), whose Sorting Intolerant from Tolerant (SIFT) score was >0.05 and PolyPhen V2 (or MutationTaster) score was <0.85. The following nine novel mutations were selected for further characterization with Sanger sequencing technology to avoid false-positive results: FNDC3B (c.455C > T:p.P152L), COL4A4 (c.3636_3637del:p.R1212fs), MPDZ (c.5015G > T:p.R1672L; c.3798dupA:p.P1267fs; and c.28G > A:p.A10T), DOCK9(c.1940C > T:p.P647L), POLG(c.127_128insGGC:p.Q43delinsRQ), IPO5(c.3019G > A:p.V1007I), and TGFBI (c.624 + 7− > A). The forward and reverse primers used in the direct polymerase chain reaction (PCR) sequencing are summarized in Table 2. The results indicated that no mutation was identified as a false-positive result with the use of Sanger sequencing technology.

The patient with the mutation FNDC3B was also detected with the mutation COL4A4 (c.3636_3637del:p.R1212fs). In addition, the patient with KC who carried the mutation DOCK9 (c.1940C > T) was also detected with a ZNF469 mutation (c.3466G > A) (the results of ZNF469 have been published [3]). The SIFT score of the ZNF469 mutation was zero, which is considered to be damaging.

**Figure 1:**
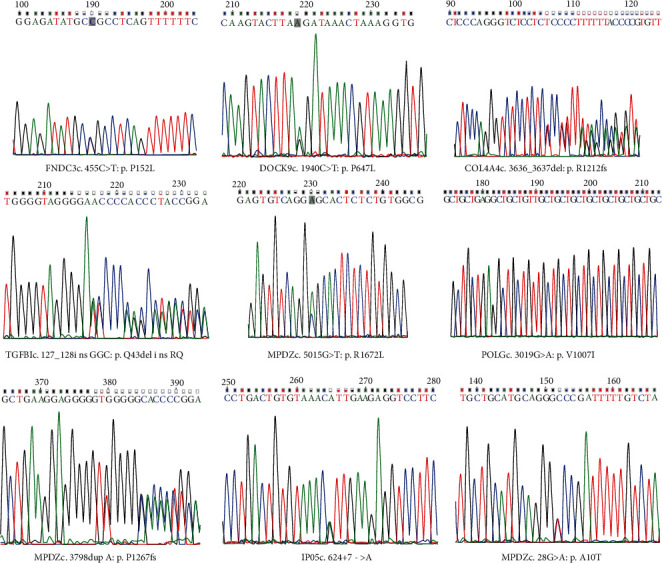
Sequence chromatogram of the nine novel mutations.

Sequencing chromatograms of the nine novel mutations in the seven genes are shown in Figure 1. All identified mutations were located in the exonic region or the region of 2 bp around the splicing junction. According to SIFT and PolyPhen V2 (or MutationTaster) scores, none of the nine mutations was classified as tolerated (Table 1).

Table 3 shows the characteristics of the eight patients with KC carrying the nine novel mutations. All patients with KC were diagnosed with bilateral KC. The mean age of the eight patients was twenty-two years old, and six patients were male. Central corneal thickness (CCT) was examined in the KC patients with videokeratography during their initial visit, and the mean values were 457 *μ*m for OD (oculus dexter) and 454 *μ*m for OS (oculus sinister).

## 4. Discussion

In the present study, nine novel mutations were identified in FNDC3B (c.455C > T:p.P152L), COL4A4 (c.3636_3637del:p.R1212fs), MPDZ (c.5015G > T:p.R1672L; c.3798dupA:p.P1267fs; and c.28G > A:p.A10T), DOCK9 (c.1940C > T:p.P647L), POLG (c.127_128insGGC:p.Q43delinsRQ), IPO5 (c.3019G > A:p.V1007I), and TGFBI (c.624 + 7− > A) in eight of the fifty-two patients with KC of Han Chinese ethnicity. The mutation in FNDC3B (c.455C > T:p.P152L) coexisted with the mutation in COL4A4 (c.3636_3637del:p.R1212fs) in one female KC patient (Table 3), and the mutation in DOCK9 (c.1940C > T:p.P647L) coexisted with a potentially damaging ZNF469 (c.3466G > A) mutation in one male KC patient. All of the mutations were found in sporadic KC cases and were absent in the results of the whole-exome sequencing (WES) data acquired. None of the nine mutations related to KC have been previously reported.

Since the first case of KC was confirmed in 1854 [9], numerous studies have been conducted to uncover the potential pathophysiology. It should be noted that positive family history was found in 6–20% of KC cases [10], and concordance between monozygotic twins was confirmed compared with dizygotic twins, which suggests that genetic factors are dominant components in KC etiology.

As is well known, the human cornea is rich in extracellular matrix (ECM), which maintains corneal transparency and biomechanical strength [11]. Through medical biochemistry techniques — such as immunostaining technology — and proteomics, different abnormalities related to varying risk genes in the expression of ECM components were identified in KC patients [12]. To date, more than ten KC-related genes have been identified as related to ECM component abnormality [12–15]. In the present study, two KC-related mutations were identified in two well-known ECM-associated genes (TGFBI and COL4A4). In KC patients, Bykhovskaya et al. identified a significantly unregulated transcript of the gene TGFBI, which was reported as related to the downregulation of collagen genes (such as COL5A1, coding for collagen V) in the ECM and caused decreased CCT [12]. TGFBI encodes the TGF*β*-induced protein and works as a transcription regulator; this extracellular protein can also mediate cell adhesion to collagen, laminin, fibronectin, and proteoglycans [16]. In this study, the coding region of TGFBI was screened, and one novel TGFBI mutation (c.624 + 7− > A) was identified, which further confirmed that TGFBI might be a risk gene for KC patients in China. Collagens are the main components of ECM, and the type IV collagen family (COL4A1 to COL4A6) consists of the corneal basement membrane [17]. The COL4A4 gene was expressed by the central corneal epithelium. With respect to the function of collagen IV, COL4A4 was also suggested to be a candidate risk gene for KC [18, 19]. In this study, one COL4A4 (c.3636_3637del:p.R1212fs) that has never been reported in Chinese KC patients was identified.

Recent studies show that part of the genes involved in KC development are related to CCT. In the present study, FNDC3B and MPDZ are widely thought of as CCT-related genes, and progressive corneal thinning is known to be a feature of the pathophysiology of KC. Lu et al. reported that rs4894535 in FNDC3B and rs1324183 in MPDZ led to decreased CCT with GWAS [20], and the results were replicated in studies by Hao and Sahebjada [21, 22]. In the present study, one novel FNDC3B (c.455C > T:p.P152L) mutation and three MPDZ (c.5015G > T:p.R1672L; c.3798dupA:p.P1267fs; and c.28G > A:p.A10T) mutations were identified in Chinese KC patients; this was consistent with former studies and provided strong genetic evidence that variants in these two genes lead to increased susceptibility to KC. Nevertheless, the role of FNDC3B and MPDZ in KC remains elusive.

DOCK9 variants were first indicated to be related to KC risk in an Ecuadorian family [23]. Former fundamental studies have shown that DOCK9 could work as an activator of the cell division cycle and regulate the wound-repairing process in the human cornea [23]. With next-generation sequencing technology, this study's KC cohort found one novel mutation (c.1940C > T:p.P647L) that was located in the exon region in DOCK9, and the mutation was predicted to be damaging by SIFT.

IPO5, which encodes a member of the kariopherin superfamily and is involved in protein nuclear transport [24, 25], was also screened in our study. One novel mutation (c.3019G > A:p.V1007I) was indicated to be related to KC in this Chinese KC cohort. Consistent with us, Justyna et al. also reported IPO5 mutations in Polish KC patients [26].

We also screened the coding region of POLG, which encodes the only DNA polymerase present in mammalian mitochondria [27, 28]. Wojcik et al. provided strong evidence that the POLG gene might play a vital role in KC pathogenesis and determining the risk of this corneal disease [29]. However, genetic studies about the relationship between KC and POLG are limited. The results of this study provide new evidence that supports Wojcik's conclusion.

This study had some limitations. First, the limited sample size influenced the significance of the present study. Secondly, just the coding region of the identified genes was screened, while deep intronic substitutions could also induce KC formation. Thirdly, negative family history indicated that all involved patients were sporadic. Thus, it is difficult to define the genetic pattern of novel mutations. In our view, the absence of these mutations in the majority of Chinese KC patients suggests that their role is not dominant, and the phenotype of the next generation would help define the genetic pattern. Fourthly, the present study examined KC-related genes reported before December 31, 2014, and the additional KC-related genomic loci identified by several genome-wide association studies conducted after that may not have been included, which may have resulted in the incomplete coverage of the loci in this study [30–33].

The exact etiology of KC remains elusive. Our study screened sixteen reported KC genes and identified nine novel mutations in FNDC3B, COL4A4, MPDZ, DOCK9, POLG, IPO5, and TGFBI, and the results should be further confirmed by well-powered GWAS screening of Han Chinese patients with KC. Further research should be designed to uncover the potential role of the novel mutations in KC etiology by analysing the corneal characteristics of genetic manipulation in animal models and corneal materials obtained from keratoplasty surgeries of mutation carriers.

## Figures and Tables

**Table 1:** All variants identified in KC patients in the present study.

Gene	Nucleotide change	Amino acid change	SNP ID	Frequency (1KG project)	Gene region	Mutation effect	SIFT score	PolyPhen V2 score	MutationTaster
NFIB	c.196C > G	p.P66A	rs140030018	0.00219649	Exonic	Nonsynonymous	0.14	0.999	
LIG3	c.1626G > T	p.K542N			Exonic	Nonsynonymous	0.06	0.573	
XRCC1	c.1141G > A	p.V381M	rs2271980		Exonic	Nonsynonymous	0	0.977	
TF	c.521C > T	p.S174L	rs150679929	0.00159744	Exonic	Nonsynonymous	0.03	0.678	
ZEB1	c.2998G > C	p.E1000Q			Exonic	Nonsynonymous	0.08	0.293	
NEIL1	c.833C > T	p.T278I	rs142213781	0.000599042	Exonic	Nonsynonymous	0	0.998	
FNDC3B	c.455C > T	p.P152L			Exonic	Nonsynonymous	0	0.963	1
FNDC3B	c.1438G > A	p.A480T	rs201974179	0.000599042	Exonic	Nonsynonymous	0.15	0.17	
COL4A4	c.3636_3637	del:p.R1212fs			Exonic	Frameshift deletion			
MPDZ	c.5015G > T	p.R1672L			Exonic	Nonsynonymous	0		
MPDZ	c.3798dupA	p.P1267fs			Exonic	Frameshift insertion			
MPDZ	c.394G > A	p.G132S	rs201101621	0.000399361	Exonic	Nonsynonymous	0.03		
MPDZ	c.28G > A	p.A10T			Exonic	Nonsynonymous	0.05		
DOCK9	c.1940C > T	p.P647L			Exonic	Nonsynonymous	0		
TGFBI	c.624 + 7− > A				Splicing				
PARP1	c.2440A > G	p.I814V	rs200630156	0.000798722	Exonic	Nonsynonymous	0.21	0.151	
PARP1	c.114G > A	p.M38I			Exonic	Nonsynonymous	0.09	0.646	
PARP1	c.14C > G	p.S5W	rs201256399	0.00119808	Exonic	Nonsynonymous	0	0.299	
POLG	c.3139C > T	p.R1047W	rs181860632	0.000399361	Exonic	Nonsynonymous	0.02	1	
POLG	c.2890C > T	p.R964C	rs201477273	0.00299521	Exonic	Nonsynonymous	0	1	
POLG	c.127_128insGGC	p.Q43delinsRQ			Exonic	Frameshift insertion			
IPO5	c.3019G > A	p.V1007I			Exonic	Nonsynonymous	0.03	0.006	0.994
FASLG	c.280T > G	p.L94V	rs56302117	0.00259585	Exonic	Nonsynonymous	0	0.778	
IL1B	c.275C > T	p.T92I	rs200401035	0.000199681	Exonic	Nonsynonymous	0.01	0.766	

**Table 2:** Sequences of primers used in this study.

Gene	Mutations	Primer name	Fragment size (bp)	Primer sequence
FNDC3B	c.455C > T:p.P152L	6F	417	TGCGTTCTTTCCTTTTGTGTT
		6R		GCTAGGAATTCCCCATGAGTC

COL4A4	c.3636_3637del:p.R1212fs	7F	324	TCCTCATTGCATTTGGAAGGT
		7R		GCAACCAGTTGTTGGTGTCTG

MPDZ	c.5015G > T:p.R1672L	12F	484	GTTCCTTAGTGAGGGGGCTAA
		12R		AATGCCTGTTTTTGACCTTCA

MPDZ	c.3798dupA:p.P1267fs	9F	494	GAGACACAGAGTGGCTGATCC
		9R		ACAAACCCACTGCATTCTTTT

MPDZ	c.28G > A:p.A10T	13F	345	CCTGCTTGGGTGAATGATGTC
		13R		GGGTCCGGCCTACTGTTTTT

DOCK9	c.1940C > T:p.P647L	17F	353	CAAACCTTAAGGGGCTGAGAG
		17R		CCTGCAGTAAAACTCCCATCA

POLG	c.127_128insGGC:p.Q43delinsRQ	11F	425	CAGCTCCACGTCGGGCAAGG
		11R		GCCCAAAGCCAGGTGTTCTGACTCC

IPO5	c.3019G > A:p.V1007I	8F	445	CCCTCTGTTGCCATTCTGTAA
		8R		AGGAAGCACTGTGGAGGAGA

TGFBI	c.624 + 7− > A	19F	402	CAGAGTTGCAAGGACCCATCT
		19R		GCAAAATGTGGGTTCCACAAG

**Table 3:** The characteristics of 8 patients carrying the nine novel mutations.

Subject	Gene	Age at detection	Gender	Eye	Keratoplasty	Central thinnest corneal thickness (OD/OS, *μ*m)
1	FNDC3B COL4A4	24	Female	OU	No	464/459
2	IPO5	17	Male	OU	No	457/479
3	MPDZ	21	Male	OU	No	405/370
4	POLG	23	Male	OU	No	459/458
5	MPDZ	16	Female	OU	No	426/456
6	MPDZ	32	Male	OU	No	532/518
7	DOCK9 ZNF469	17	Male	OU	No	414/386
8	TGFBI	24	Male	OU	No	502/506

## Data Availability

The datasets used and/or analysed during the current study are available from the corresponding author on reasonable request.

## Supplementary Materials

Supplementary Materials Compare with the KC-related mutations reported in the PubMed database from January 1, 2015, to May 31, 2021.
