# Supplementary material for: Novel Mutations Identified in the Chinese Han Population with Keratoconus by Next-Generation Sequencing
Source: J Ophthalmol. 2022 Feb 10;2022:9991910. doi: 10.1155/2022/9991910 (PMC8853779; doi:10.1155/2022/9991910)
Supplement: Supplementary Materials — Compare with the KC-related mutations reported in the PubMed database from January 1, 2015, to May 31, 2021. [file 9991910.f1.docx]

Compare with the KC-related mutations reported in the pubmed database from ***January 1, 2015 to May 31, 2021***

c.455C>T:p.P152L in ***FNDC3B***

<https://doi.org/10.1038/s42003-021-01784-0>

<https://pubmed.ncbi.nlm.nih.gov/25675348/>

c.3636_3637del:p.R1212fs in ***COL4A4***

<https://www.ncbi.nlm.nih.gov/pmc/articles/PMC7431712/>

<https://pubmed.ncbi.nlm.nih.gov/25651396/>

c.5015G>T:p.R1672L;

c.3798dupA:p.P1267fs;

c.28G>A:p.A10T in ***MPDZ***

<https://pubmed.ncbi.nlm.nih.gov/30002070/>

c.624+7->A in ***TGFBI***

[*https://pubmed.ncbi.nlm.nih.gov/28567551/*](https://pubmed.ncbi.nlm.nih.gov/28567551/)

**The above genes have been reported but the mutations are different.**

c.1940C>T:p.P647L in ***DOCK9***

c.127_128insGGC:p.Q43delinsRQ in ***POLG***

c.3019G>A:p.V1007I in ***IPO5***,

**The above genes have not been reported**
